# Human Activities and Climate Separately Influence the Global Dispersal and Colonization Potential of *Lantana camara* L.

**DOI:** 10.3390/biology15100775

**Published:** 2026-05-13

**Authors:** Honglin Guo, Yuanhai Wang, Haohao Wen, Liqun Long, Mu Duan, Yuanxin Wang, Zhaochen Xu, Jingjing Du, Dong Jia

**Affiliations:** 1 Shanxi Fenhe Plain Farmland Shelterbelt Ecosystem Observatory and Research Station, Shanxi Key Laboratory of Bioagent Utilization and Eco-Pesticide Innovation, College of Plant Protection, Shanxi Agricultural University, Taigu, Jinzhong 030801, China; 2 State Key Laboratory of Animal Biodiversity Conservation and Integrated Pest Management, Institute of Zoology, Chinese Academy of Sciences, Beijing 100101, China; 3 Anhui Provincial Key Laboratory of Forest Resources and Silviculture, School of Forestry & Landscape Architecture, Anhui Agricultural University, Hefei 230036, China; 4 College of Life Science, Hebei University, Baoding 071002, China

**Keywords:** *L. camara*, species distribution models, biological invasion, human activity, climate change, suitable habitat prediction

## Abstract

The global invasion of the shrub *L. camara* poses a significant threat to ecosystems. Understanding the roles of human activity and climate in driving its spread is crucial for management. This study aimed to quantify its global invasion dynamics, identify key drivers, and predict future distribution shifts. We constructed a high-precision ensemble species distribution model by integrating historical global occurrence records, multi-source environmental variables (climate and human activity indices), and future climate scenarios (SSP1-2.6 and SSP5-8.5). The global invasion showed a clear four-stage acceleration pattern (1900–1960, 1961–1980, 1981–2000, and 2001–2025). Variable importance and response curve analysis revealed a two-phase “dispersal–colonization” mechanism: human activities (e.g., gross domestic product) acted as a “dispersal amplifier,” while a climatic factor (isothermality) served as a critical “colonization filter.” Under two future climate scenarios assuming unchanged human activity patterns, the potential suitable habitat of *L. camara* exhibits structural changes while maintaining stable total area. The highly suitable areas continue to shrink, with nearly half the area lost by the end of the century under the high-emission SSP5-8.5 pathway, while low-suitability zones expand significantly—yet the overall suitable habitat remains stable. Under SSP1-2.6, structural changes in suitable habitats occur more gradually. The study clarifies the distinct roles of human activity and climate in the invasion process, providing a scientific basis for differentiated global risk management strategies targeting dispersal pathways and colonization thresholds.

## Simple Summary

This study addresses the increasing threat of the invasive *L. camara* shrub to ecosystems and biodiversity worldwide. We combined its past spread records with environmental data and future climate projections to build a predictive model. Our goal was to map its global invasion, understand what drives it, and forecast where it might spread next. The findings show that its invasion has accelerated in stages, now covering many tropical and subtropical areas. In certain situations, human activities, like economic development, primarily act as a “dispersal amplifier,” helping plants reach new regions. In contrast, climate conditions, particularly temperature stability, serve as a “colonization filter,” determining where it can survive. Looking ahead, while the highest-suitability habitats are projected to shrink substantially, the plant’s overall suitable range is predicted to remain relatively stable in total area but undergo significant internal restructuring—with a marked expansion of lower-suitability areas. This shift suggests a diffusion of invasion risk into wider marginal zones rather than a simple range expansion. This research clarifies the distinct roles of human and climate factors in biological invasions, offering vital insights for global management and local prevention strategies.

## 1. Introduction

Biological invasions represent one of the most severe ecological challenges in the era of globalization, posing substantial threats to biodiversity, ecosystem functioning, and socioeconomic development in invaded regions [1,2]. Against the backdrop of the Anthropocene [3], climate change and human activities—two major global drivers—are jointly reshaping the geographical distribution patterns of species at an unprecedented intensity [4,5]. By modifying environmental conditions such as temperature and precipitation, climate change directly influences the physiological tolerance thresholds of species, creating potential new suitable habitats [6,7,8]. Concurrently, intense human activities, including international trade, transportation network expansion, and land-use change, have significantly weakened geographical barriers [7], providing efficient dispersal pathways for alien species and generating disturbed environments favorable for their establishment and spread [9,10]. These two drivers interact and reinforce each other; however, their relative contributions and mechanisms across different stages of invasion (e.g., initial dispersal and subsequent colonization) remain key scientific questions in invasion ecology.

*L. camara* belongs to the Verbenaceae family and is native to tropical America. It has now become one of the most widely distributed invasive shrubs globally [11]. In the early 17th century, it was introduced to Europe as an ornamental plant by Dutch explorers from Brazil, widely cultivated as a horticultural plant in Europe, and introduced to many parts of the world through colonial and trade activities. This species exhibits strong adaptability and rapid growth, with significant allelopathic effects [12]. It tends to form monospecific stands, outcompeting native plants, reducing regional biodiversity, and causing substantial economic losses to agricultural, forestry, and livestock production [13,14]. This species has a strong dispersal ability. The seeds of *L. camara* are dispersed over long distances via consumption by birds and other animals. Once germinated in a suitable habitat, the plant can rapidly expand locally through its creeping branches. Since its introduction as an ornamental plant in the 19th century, *L. camara* has successfully invaded more than 60 countries, becoming particularly rampant in tropical and subtropical regions [15]. Although its invasive impacts have been widely recognized, systematic and integrated studies are still lacking to quantitatively characterize the spatiotemporal dynamics of its global invasion, identify the key factors driving its dispersal and colonization, and systematically predict its potential distribution risks under future climate change scenarios.

In recent years, species distribution models have become important tools for predicting the potential distribution of species and identifying environmental drivers [16,17]. However, most studies have focused either solely on climatic variables or used only a single human activity index [18,19,20], making it difficult to fully quantify and compare the distinct roles of climate and human activities across the multi-stage invasion process. Meanwhile, existing studies have mostly focused on changes in the total area of suitable habitats [21], with insufficient attention to spatial shifts and structural changes among suitability classes (high, medium, and low suitability), which are critical for refined invasion risk assessment.

To address these research gaps and test the core hypothesis that human activity and climate play distinct roles as a “dispersal amplifier” and “colonization filter”, respectively, leading to structural changes in future suitable habitats, this study integrates global historical invasion records, multi-source environmental variables, and future climate scenarios. It aims to (1) systematically reveal the global spread dynamics and current distribution pattern of *L. camara* since the early 20th century; (2) quantitatively compare the influences of human activities and climatic variables on its distribution and interpret the underlying mechanisms; and (3) predict the structural changes in its global suitable habitat by the end of the 21st century under different socioeconomic pathways. Achieving these objectives is crucial, as clarifying the invasion dynamics provides the foundation for phased management strategies, distinguishing the stage-specific drivers enables differentiated measures targeting dispersal and colonization, and predicting habitat structural shifts supports more refined regional risk assessment and resource allocation, ultimately offering a scientific basis for global risk management.

## 2. Materials and Methods

### 2.1. Data Sources

**Figure A1:**
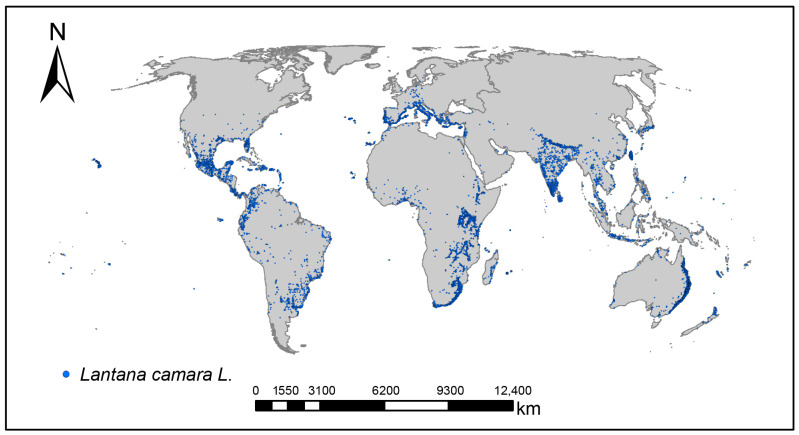
Filtered and diluted global distribution points of *L. camara*.

Distribution data for *L. camara* were obtained from the Global Biodiversity Information Facility (GBIF) database. A total of 99,787 occurrence records were collected (https://doi.org/10.15468/dl.z8etzj, accessed on 29 December 2025). All occurrence records were verified, and data with the following geographic characteristics were removed: records located in national capitals, provincial centers, research institutions, museums, and marine areas; records with fully duplicated latitude and longitude; and records with coordinates (0, 0). Subsequently, the collected occurrence points were thinned using the R packages sf and terra(R version 4.5.2), retaining only one occurrence point per grid cell at a spatial resolution of 2.5 arc-minutes [22,23]. After processing, 17,237 valid occurrence points were retained (Appendix A, Figure A1).

### 2.2. Collection and Processing of Environmental Variables

Bioclimatic data were obtained from the World Clim database (http://www.worldclim.org/, accessed on 25 January 2026). This dataset provides historical bioclimatic data for 1970–2000, as well as climate projections for every 20-year period from 2041 to 2100 (i.e., 2041–2060, 2061–2080, and 2081–2100) under different socioeconomic scenarios. We also incorporated climate projection data under the Shared Socioeconomic Pathways (SSPs) from the Coupled Model Intercomparison Project of the IPCC Sixth Assessment Report to account for future climate change [24,25]. Specifically, the greenhouse gas emission scenarios SSP1-2.6, SSP2-4.5, SSP3-7.0, and SSP5-8.5 represent low, medium, medium-high, and high radiative forcing levels, respectively [26,27]. This study selects two representative scenarios, SSP1-2.6 (low-carbon sustainable pathway) and SSP5-8.5 (high-carbon fossil fuel pathway), aiming to cover the spectrum of future societal development and climate forcing from the most optimistic to the most pessimistic. The objectives are as follows: (1) To assess the sensitivity of invasion risk to the intensity of climate change: By contrasting extreme scenarios, the study reveals the range and patterns of *L. camara*’s potential distribution in response to different levels of climate forcing. (2) To provide a comprehensive risk management perspective: The results under SSP1-2.6 represent the ecological consequences that may emerge under strong global climate mitigation policies, serving as a reference baseline for formulating long-term biological invasion prevention and control strategies under ideal objectives. Although its realization faces challenges, the predictions under this scenario still hold significant scientific value for understanding invasion dynamics under optimal mitigation efforts. To improve data reliability, in addition to bioclimatic variables, we also integrated three commonly used human impact indices: gross domestic product (https://zenodo.org/records/16741980, accessed on 30 December 2025) [28], road density (https://zenodo.org/records/6420961, accessed on 30 December 2025) [29], and human footprint index (https://www.x-mol.com/groups/li_xuecao/news/48145, accessed on 30 December 2025) [30]. All raster data were resampled to a uniform spatial resolution of 2.5 arc-minutes to ensure spatial consistency among all indicators.

**Figure A2:**
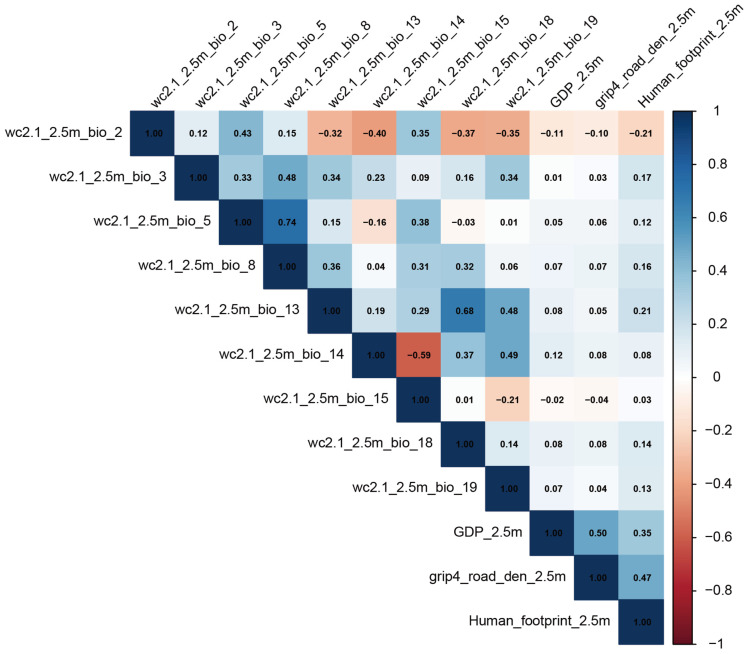
Spearman correlation matrix of environmental variables (|r| < 0.8).

Variable screening for the 19 climatic factors and human activity variables was performed using Spearman’s correlation coefficient to address multicollinearity. Variables with a correlation coefficient greater than 0.8 were regarded as highly correlated and removed. Finally, 12 environmental variables with significant effects on the distribution of *L. camara* were selected: GDP (Bio20), road density (Bio21), human footprint index (Bio22), mean diurnal temperature range (Bio2), isothermality (Bio3), maximum temperature of the warmest month (Bio5), mean temperature of the wettest quarter (Bio8), precipitation of the wettest month (Bio13), precipitation of the driest month (Bio14), precipitation coefficient of variation (Bio15), precipitation of the warmest quarter (Bio18), and precipitation of the coldest quarter (Bio19) (Appendix A, Figure A2).

### 2.3. Species Distribution Modeling

Pseudo-absence points were generated by random sampling based on species occurrence data combined with climatic and human activity variables. Specifically, 17,000 pseudo-absence points were randomly generated across global land areas. An ensemble species distribution model was then constructed using the R (version 4.3.2) package biomod2 [31]. First, 70% of the *L. camara* occurrence data (presence–absence records) were randomly selected as the training set, with the remaining 30% used for model evaluation. Subsequently, species distribution predictions were conducted using a high-performance computing cluster.

Ten commonly used high-performance species distribution model algorithms were considered in the ensemble model: Random Forest (RF), Multivariate Adaptive Regression Splines (MARS), Maximum Entropy (MaxEnt), Generalized Additive Model (GAM), Generalized Linear Model (GLM), Gradient Boosting Machine (GBM), Classification Tree Analysis (CTA), Artificial Neural Network (ANN), Surface Range Envelope (SRE), and Flexible Discriminant Analysis (FDA) [32].

Subsequently, robustness evaluation and three replicate cross-validations were conducted for the 10 species distribution models. The robustness of the 10 models (model selection) was calibrated and validated using the True Skill Statistic (TSS) and the AUC (Area Under the ROC Curve) value calculated based on the ROC (Receiver Operating Characteristic Curve) (Appendix A, Table A1). Values ranged from 0.5 to 1, with higher values indicating higher model prediction accuracy. The three models with the top-ranked TSS and AUC values (RF, GAM, and GBM) were then selected and used to construct ensemble models via EMmean (Mean), EMca (Committee Averaging), and EMwmean (Weighted Mean), respectively [33]. The performance of the integrated model is evaluated based on its performance on the calibration dataset to measure its ability to integrate information from individual models (Appendix A, Table A2), and EMwmean was finally chosen for subsequent analysis.

Next, permutation-based variable importance assessment was used to quantify the relative contributions of climatic and human activity variables to predicting the potentially suitable habitats of *L. camara* [34]. This method calculated the decline in model predictive performance by randomly shuffling the values of one variable at a time after model training, which served as the importance index of that variable. After the calculations, response curves of the main variables were generated to reveal their effects on model outputs across different value ranges.

The potential suitable habitat map of *L. camara* under the current climate had a value range from 0 to 1. These values were reclassified into four potential habitat categories based on the natural breaks method: highly suitable (0.638–1), moderately suitable (0.354–0.638), marginally suitable (0.127–0.354), and unsuitable (<0.127). After modeling the spatial extent of suitable habitats for *L. camara* using current climate data, modeling and prediction were performed for six climate time scenarios across three periods (2050s, 2070s, and 2090s) under two future climate scenarios (SSP1-2.6 and SSP5-8.5) to project changes in suitable habitat ranges [35]. To assess the uncertainty of our ensemble predictions, we calculated the standard deviation of the continuous suitability predictions (0–1) from the three constituent models (RF, GAM, and GBM) for each time period and scenario. The spatial mean of these standard deviation values across all scenarios was 0.053, providing a quantitative measure of overall model prediction uncertainty.

## 3. Results

### 3.1. Global Spread Dynamics and Current Distribution Status of L. camara

**Figure 1:**
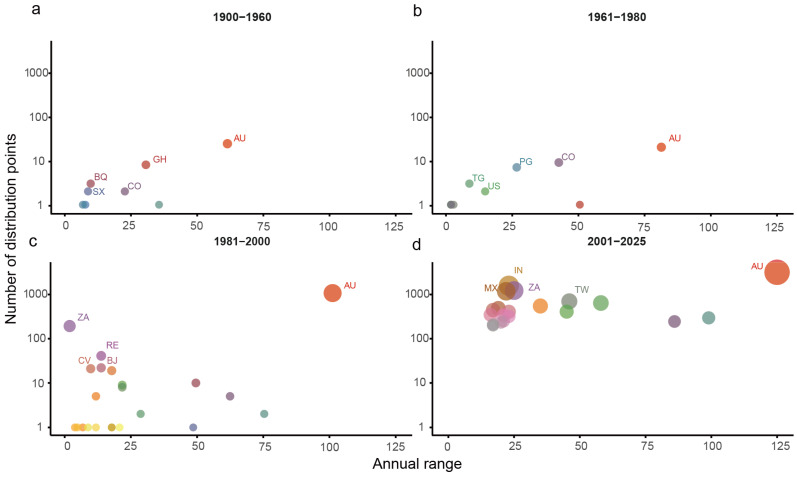
Global spread dynamics of *L. camara*. The horizontal axis represents the duration of *L. camara*’s invasion in each country, while the vertical axis shows the number of distribution points for *L. camara* during each period. The size of the points also indicates the number of distribution points, with larger sizes representing higher counts. The top five countries (Color differentiation) with the highest number of distribution points in each period are labeled on the graph, using ISO 3166-1 (Alpha-2) standard abbreviations [36]. (**a**) 1900–1960 spread dynamics; (**b**) 1961–1980 spread dynamics; (**c**) 1981–2000 spread dynamics; (**d**) 2001–2025 spread dynamics.

Analysis of historical invasion records shows that the global spread of *L. camara* displays obvious phased and accelerated characteristics. Its dispersal process can be divided into four stages: Initial introduction and colonization stage (1900–1960): The invasion range was limited to eight countries/regions, represented by Australia (1900) and Ghana (1931), showing a scattered point-like distribution (Figure 1a). Local spread stage (1961–1980): The distribution expanded to 10 countries/regions, adding new frontier areas such as the United States (1967) and Taiwan, China (1979) (Figure 1b). Regional outbreak stage (1981–2000): The invasion process accelerated significantly, with recorded sites expanding to 24 countries/regions. Population outbreaks occurred in South Africa, Australia and other regions, and the species began to spread to more areas of Asia and Africa (Figure 1c). Global expansion stage (2001–2025): The invasion entered an explosive phase, with recorded countries/regions exceeding 120. India, South Africa, Mexico, China, Spain, Brazil and other regions each recorded more than 400 sites, becoming core regions of global invasion (Figure 1d). In many areas, large-scale populations were established within a short time after the first record in the early 21st century. By 2025, *L. camara* had evolved from localized colonization in the early 20th century to a widespread invasion pattern across tropical, subtropical and some temperate coastal regions worldwide.

### 3.2. Comparison of the Effects of Human Activities and Climate Change on Habitat Suitability of L. camara

Variable importance analysis revealed the key environmental factors affecting the potential distribution of *L. camara*. The two importance indices from the Random Forest model showed significant differences (Table 1). Mean Decrease in Accuracy (MDA) based on model accuracy indicated that isothermality was the most important factor affecting prediction accuracy (MDA = 88.32), with a contribution much higher than that of other variables. This was followed by road density (MDA = 46.90) and GDP (MDA = 53.82), which represent human activities. In contrast, the Mean Decrease in Gini (MDI) based on node impurity reduction showed a different ranking: GDP showed the most prominent importance (MDI = 229.58), followed by the human footprint index (MDI = 156.68) and precipitation of the wettest month (MDI = 97.57), while isothermality ranked fourth (MDI = 123.62). The discrepancy between these two indices suggests that human activity variables are frequently and effectively used in model construction rules, whereas climatic factors (especially isothermality) make irreplaceable and unique contributions to maintaining the robustness of model predictions.

The variable importance results of the ensemble species distribution model were highly consistent with the MDA ranking of Random Forest (Table 2). In the ensemble model, isothermality had the highest contribution rate (26.22%), further confirming its key role as the core environmental constraint. Human activity variables such as human footprint index (5.77%) and GDP (3.97%) also showed important contributions, but their importance values were much lower than isothermality. Other bioclimatic variables, such as mean temperature of the wettest quarter (5.09%) and maximum temperature of the warmest month (2.32%), also had certain effects, while the contribution rates of all precipitation variables were generally low (<2%). Overall, the model analyses consistently indicate that isothermality is the primary limiting factor controlling the global potential distribution of *L. camara*, and human activity intensity is another important driving force shaping its distribution pattern.

**Figure 2:**
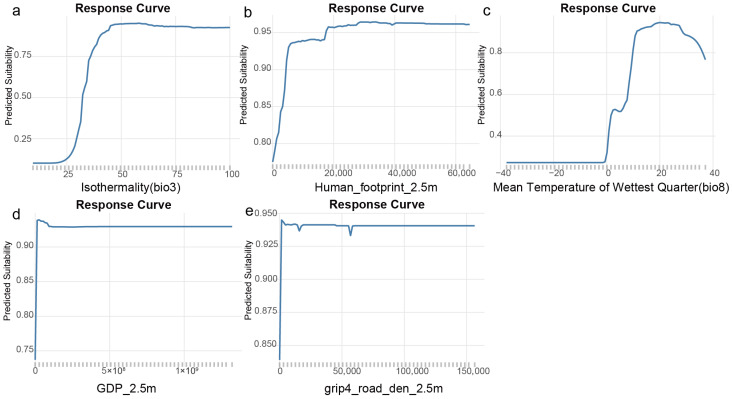
Response curves of high-contribution environmental factors for the global distribution of *L. camara*: (**a**) isothermality (bio3, a unitless ratio reflecting temperature stability), (**b**) Human_footprint_2.5m (a unitless composite index), (**c**) mean temperature of wettest quarter (bio8, in °C), (**d**) GDP_2.5m (in USD), and (**e**) grip4_road_den_2.5m (in m/km^2^).

The response curves clearly illustrate the quantitative effects of the top five environmental variables with the highest contribution rates on the potential suitability of *L. camara* (Figure 2). The curve shapes reveal distinct modes of action for different types of factors, and their morphological differences mechanistically confirm the results of the variable importance analysis. Threshold effect of climatic factors: As the most critical climatic variable, isothermality exhibits a typical response pattern of “low initial level–steep rise–plateau” (Figure 2a). When isothermality is low (i.e., large seasonal temperature fluctuations), the predicted suitability is close to zero. Once the threshold (approximately 25) is exceeded, suitability rises sharply to a high level and stabilizes. This indicates that isothermality sets a rigid minimum tolerance threshold for the survival of *L. camara*, acting as the core limiting factor determining its successful colonization. The response curve of another important climatic variable, mean temperature of the wettest quarter (Figure 2c), also shows a nonlinear increasing trend, with an optimal range in the moderate temperature interval (10–25 °C). Gain effect of human activity factors: Unlike climatic factors, the response curves of human activity variables start at a relatively high background suitability. The curve for GDP (Figure 2b) shows a pattern of “high initial level–steep rise–plateau”. Even at extremely low GDP levels, the predicted suitability remains moderate (approximately 0.4) and then increases rapidly to saturation with rising GDP. This suggests that human economic activity is not a “permissive” condition for the colonization of *L. camara* but rather acts as a powerful “dispersal amplifier” that significantly elevates its colonization success rate (predicted suitability) from the background level to near the theoretical maximum within climatically suitable regions. The response curves of human footprint index and road density (Figure 2d,e) also show similar saturating growth patterns, further corroborating the dominant role of human activities in promoting population establishment and dispersal.

### 3.3. Trends in Current and Future Suitable Habitats for L. camara

**Figure 3:**
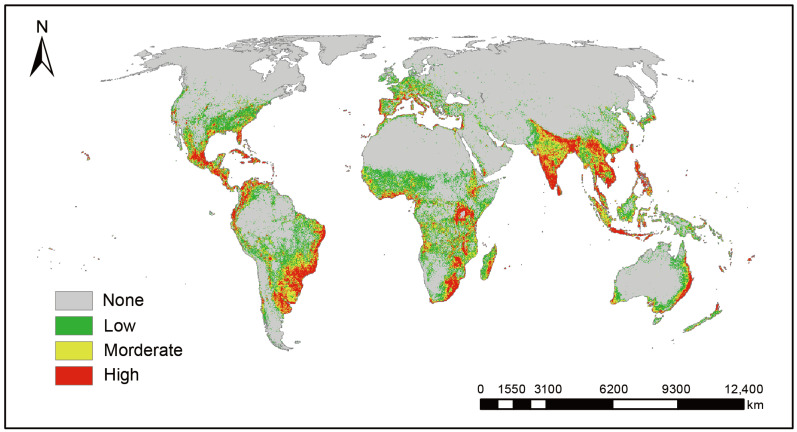
Current suitable habitat distribution of *L. camara*.

Under current climatic conditions, the total global potential suitable habitat area of *L. camara* is approximately 4.245 million square kilometers (Table 3). Among them, the areas of high-suitability habitat, medium-suitability habitat, and low-suitability habitat are 1.026, 1.242, and 1.977 million square kilometers, accounting for 24.2%, 29.3%, and 46.6% of the total suitable habitat area, respectively. The suitable habitats of *L. camara* are mainly distributed in Central America, southeastern South America, southern Africa, Southeast Asia, and eastern Oceania (Figure 3).

**Figure 4:**
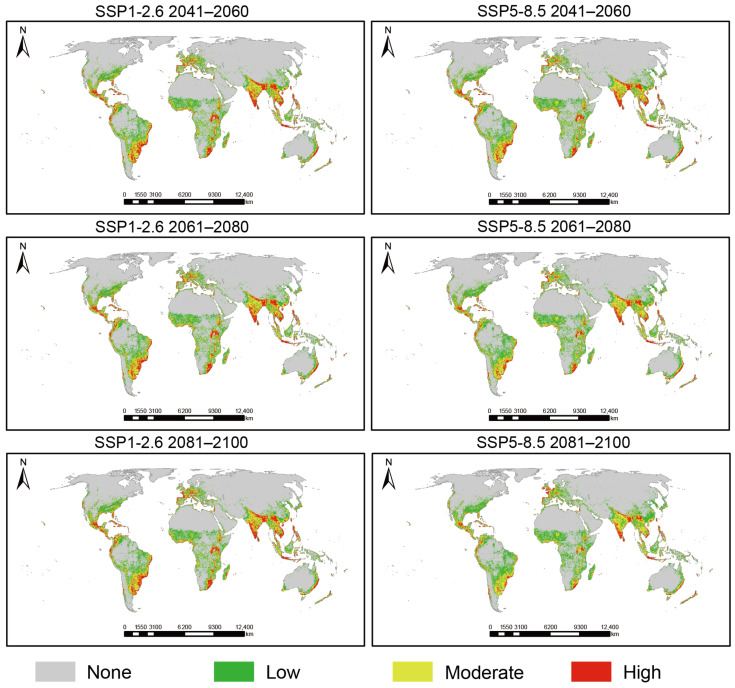
Suitable habitat distribution of *L. camara* in future periods.

Projections under different future Shared Socioeconomic Pathways (SSP1-2.6 and SSP5-8.5) show that the structural changes in suitable habitats of *L. camara* exhibit a consistent direction but differ in magnitude among scenarios (Table 3, Figure 4). High-suitability habitats continue to shrink; the area of high-suitability habitat shows a clear declining trend in all future periods and scenarios. This contraction is particularly pronounced under the high-emission pathway (SSP5-8.5). By the end of this century (2081–2100), under the SSP1-2.6 pathway, the high-suitability habitat area is projected to decrease to 1.093 million km^2^ (a reduction of 6.5% from the current 1.026 million km^2^); under the SSP5-8.5 pathway, it drops sharply to 0.686 million km^2^, representing a substantial loss of 33.1%. Medium- and low-suitability habitats show divergent trends; in contrast to the shrinkage of high-suitability areas, the area of low-suitability habitat is projected to expand consistently across all scenarios. Under the SSP5-8.5-2090s scenario, it increases to 2.197 million km^2^ (an increase of 11.1% from the current 1.977 million km^2^). However, the medium-suitability habitat area does not show a consistent “increasing trend”; it fluctuates slightly across scenarios, with a net change under SSP5-8.5-2090s of +0.192 million km^2^ (a 15.5% increase) compared to the current 1.242 million km^2^. Total suitable area remains stable with significant internal restructuring; despite the marked loss of high-suitability habitat, the total global potential suitable area for *L. camara* remains highly stable under all future projections, fluctuating minimally around 4.2–4.3 million km^2^. The slight variation (e.g., to 4.317 million km^2^ under SSP5-8.5-2090s) is well within the range of model prediction uncertainty. This stability masks a profound internal reorganization: the spatial pattern is characterized by the fragmentation and contraction of core (high-suitability) habitats, concurrently with the spatial connection and expansion of marginal (low-suitability) habitats.

## 4. Discussion

This study systematically analyzed the spatiotemporal dynamics, key driving mechanisms, and future trends in the global invasion of *L. camara.* The results show that its invasion process is synergistically driven by human activities and climate change, but the two play distinctly different roles in the two key stages of dispersal and colonization, jointly shaping its current and future distribution patterns.

Our study quantitatively reveals that the global invasion of *L. camara* from the 20th century to the present exhibits a clear four-stage accelerated pattern. These dynamics are highly synchronized with the phasic deepening of globalization. The early punctate introduction and local spread were associated with horticultural trade and interregional material circulation during the colonial era [37]. The explosive global expansion from the late 20th century to the present, however, is closely coupled with characteristics of the Anthropocene, especially the acceleration of global economic integration, the sharp increase in the density of international trade networks, and the exponential improvement in the efficiency of air and maritime logistics [38,39]. This confirms the core hypothesis that human activities act as the primary dispersal vector for invasive species. The high overlap between invasion hotspots (e.g., India, South Africa, and the southeastern coast of China) and major global ports, transportation hubs, and economically active areas further supports that human-mediated dispersal is the primary driving force for breaking geographical barriers and achieving transcontinental jump dispersal.

One of the most important findings of this study is that the combination of variable importance analysis and response curves mechanistically distinguishes the differential roles of human activities and climatic factors. In the Random Forest model, the high MDI values of human activity variables contrast sharply with the high MDA values of climatic variables (especially isothermality). MDI reflects the effectiveness of variables in constructing model classification rules, which corresponds to the strong statistical correlation between human activity indicators and species distribution data in space, essentially characterizing dispersal pathways and opportunities [40,41].

The strong positive correlation between GDP, road density, and invasion risk reveals the core mechanism by which socioeconomic activities drive biological invasions. As a comprehensive indicator of human activity, GDP primarily functions at two levels: First, during the introduction and dispersal stages, regions with higher GDP typically correspond to more frequent international trade, denser global shipping and aviation networks, and more active horticultural and agricultural product exchanges [39,42]. These activities directly serve as vectors for the transcontinental and cross-regional “jumps” of *L. camara* seeds and propagules, significantly increasing their chances of entering new areas [43,44]. Second, during the colonization and establishment phases, economic development often accompanies large-scale land-use changes (e.g., urbanization, agricultural reclamation, and infrastructure construction) [45]. These anthropogenic disturbances create numerous habitats resembling “niche vacancies” [46]. As an opportunistic species, *L. camara* can rapidly occupy these disturbed habitats and establish populations [47]. The “high baseline–rapid saturation” pattern observed in the GDP response curve of this model (Figure 2b) precisely confirms this: even in regions with extremely low GDP (i.e., minimal human influence), the species can still survive if the climate is suitable (moderate suitability). Meanwhile, rising GDP (representing a sharp increase in human-mediated introduction opportunities and habitat disturbance) can dramatically elevate its colonization success probability to the theoretical maximum. Thus, GDP and road density can be regarded as critical links connecting global economic drivers with local invasion risks, quantifying the “dispersal momentum” and “colonization convenience” provided by human socioeconomic activities for invasive species.

In contrast, the high contribution rate of isothermality in the ensemble model and its low initial level–steep rise response curve highlight its rigid role as a colonization filter. Isothermality reflects temperature stability, and the threshold it sets is likely a key physiological bottleneck for *L. camara* to complete its life cycle, especially to tolerate extreme low-temperature events. This finding is partly consistent with the niche conservatism hypothesis, suggesting that despite its strong invasiveness, the global distribution of *L. camara* is still deeply constrained by climatic adaptations in its native range [48,49]. Therefore, the successful invasion of *L. camara* may result from the overlap between a human-driven high-speed dispersal network and a physiologically tolerant space defined by climate: human activities determine where and how quickly it can arrive, while climatic conditions determine whether it can survive and reproduce after arrival [50].

Projections under future climate scenarios reveal a structural shift in the potential distribution of *L. camara*, characterized by the contraction of high-suitability habitats and the expansion of medium- and low-suitability habitats. This pattern of declining quality but increasing quantity carries important ecological and management implications. The shrinkage of high-suitability habitats, especially the severe loss under the SSP5-8.5 scenario, may be related to the increase in extreme temperature events and greater climate volatility in the future, which could decrease the long-term stability of populations in current core invasion areas and even trigger local population declines. However, the large-scale expansion of medium- and low-suitability habitats is even more alarming. This means that the invasion risk of *L. camara* will spread from current hotspots to a wider range of suboptimal marginal habitats. Many of these areas may be important ecological protected areas or agricultural regions that are not currently regarded as high-risk areas. Such expansion suggests that *L. camara* may have a certain capacity for niche shift, allowing it to adapt to habitats slightly beyond its current optimal range [51,52]. Therefore, future invasion risk management strategies need to shift from focusing on current high-suitability habitats to simultaneously monitoring vast medium- and low-suitability areas and preventing outbreaks into new highly suitable populations in novel regions.

This study demonstrates that the global invasion of *L. camara* is a typical microcosm of the synergistic effects of human activities and climate change in the process of globalization. Human activities construct a high-speed road network for global dispersal, while climatic conditions set entry gates for species colonization. Looking ahead, although core high-suitability habitats may shrink, the overall scope of invasion risk is expanding and becoming more dispersed. This requires us to adopt more forward-looking and differentiated management strategies: strengthening source control and interception at dispersal hotspots with intense human activities; enhancing early monitoring and rapid response at colonization frontiers driven by climate; and maintaining vigilance over continuously expanding medium- and low-suitability habitats. Only in this way can we more effectively manage this worldwide invasive species and mitigate its ecological, social, and economic impacts in the context of global change. Future research could be further deepened in the following directions: first, integrating individual or process-based models to quantitatively reveal the key ecological thresholds of “dispersal–colonization”; second, extending the driving framework of this study to other key invasive species and exploring the responses of species interaction networks under climate change. These efforts will advance biological invasion research from static prediction to dynamic regulation, providing a more solid scientific foundation for addressing biosecurity challenges under global change.

The framework of this study focuses on large-scale human activities and climatic drivers, revealing a two-stage mechanism of “dispersal–colonization.” However, the invasion process is complex. First, this study did not directly quantify biological interactions (e.g., competition with native plants and mutualistic relationships) and propagule pressure (i.e., the number and frequency of introduction events), which are local-scale processes that can significantly influence invasion success [53]. Second, while land-use change, as a key manifestation of human activity, has been partially captured through composite indicators like the human footprint index, its independent effects and dynamic processes warrant further disentanglement in future research. Lastly, although the strong constraint of isothermality supports the inference of niche conservatism, we did not conduct a direct comparison of *L. camara*’s ecological niches in its native and invaded ranges.

However, several limitations should be considered when interpreting these findings. The first is the use of static anthropogenic variables. While the selected variables (GDP, human footprint index, and road density) align temporally with the baseline period of species occurrence data, we assumed their spatial patterns would remain unchanged in future climate projections. This common simplification may overestimate future suitability in currently human-dominated areas and underestimate it in regions that become climatically suitable but currently lack anthropogenic pressure, thereby affecting the spatial accuracy of predictions and the interpretation of climate change’s relative role. Second, our correlative SDM framework does not explicitly account for dispersal limitations. The model assumes the species can reach all environmentally suitable areas within the projected time frames, which may lead to overestimation of potential distribution in geographically isolated or distant regions that are otherwise suitable. Third, spatial sampling bias in occurrence data may conflate ecological signals with collection artifacts. Records are often clustered near roads, populated areas, and other easily accessible locations, which strongly overlap with the spatial distribution of human activity variables. Consequently, the high importance attributed to these variables in the model may reflect not only the species’ genuine affinity for disturbed habitats but also the underlying bias in where it has been recorded. Finally, the interpretation of variable importance requires caution, particularly regarding metric discrepancies. The Mean Decrease in Impurity (MDI; Gini importance) is known to be biased toward correlated predictors and variables with high cardinality. Therefore, MDI may overestimate the importance of human variables.

## 5. Conclusions

Based on the integrated analysis, this study reveals a significant association between the global distribution of *L. camara* and the synergistic interplay of human activity and climate, supporting a conceptual two-phase “dispersal–colonization” framework. Statistical models indicate that proxies for human activity, such as economic intensity and road networks, are strongly linked to its presence, suggesting a role as a primary facilitator for dispersal and initial establishment in new regions. Conversely, climatic conditions, particularly temperature stability (isothermality), emerge as a key correlative filter, defining the broad environmental thresholds within which sustained colonization and spread become viable. This pattern underscores how anthropogenic and climatic factors may jointly shape invasion patterns at a global scale.

## Figures and Tables

**Table 1:** Importance analysis of environmental variables based on Random Forest.

Environmental Variable	MDA	MDI
Isothermality (bio3)	88.32	123.62
GDP (bio20)	53.82	229.58
Human Road Density (bio21)	46.9	132.86
Human Footprint Index (bio22)	40.66	156.68
Precipitation of Wettest Month (bio13)	39	97.57
Max. Temperature of Warmest Month (bio5)	36.55	44.44
Mean Temperature of Wettest Quarter (bio8)	35.42	46.23
Mean Diurnal Temperature Range (bio2)	34.53	34.6
Precipitation of Warmest Quarter (bio18)	32.26	47.17
Precipitation of Coldest Quarter (bio19)	28.56	35.9
Precipitation Seasonality (bio15)	28.49	23.03
Precipitation of Driest Month (bio14)	28.42	28.31

**Table 2:** Variable importance analysis of the ensemble species distribution model (ensemble SDM).

Environmental Variable	Contribution (%)
Isothermality (bio3)	26.22
Human_footprint_2.5min	5.77
Mean Temperature of Wettest Quarter (bio8)	5.09
GDP_2.5min	3.97
grip4_road_den_2.5min	3.04
Max. Temperature of Warmest Month (bio5)	2.32
Precipitation of Wettest Month (bio13)	1.67
Mean Diurnal Temperature Range (bio2)	1.37
Precipitation of Driest Month (bio14)	0.89
Precipitation Seasonality (bio15)	0.80
Precipitation of Coldest Quarter (bio19)	0.32
Precipitation of Warmest Quarter(bio18)	0.26

**Table 3:** Suitable areas for *L. camara* in different periods and scenarios.

Climate Scenario	Period (Year)	Unsuitable Area (10^6^ km^2^)	Low-Suitable Area (10^6^ km^2^)	Medium-Suitable Area (10^6^ km^2^)	High-Suitable Area (10^6^ km^2^)	Total Area Suitable (10^6^ km^2^)
Current	2025	91.56	19.77	12.42	10.26	42.45
ssp1-2.6	2041–2060	91.43	21.38	12.62	8.58	42.58
	2061–2080	91.38	21.63	12.63	8.38	42.64
	2081–2100	91.23	21.60	12.67	8.52	42.79
ssp5-8.5	2041–2060	91.84	21.79	12.65	7.74	42.18
	2061–2080	91.73	23.24	12.45	6.60	42.39
	2081–2100	91.93	24.67	12.21	5.20	42.08

**Figure A3:**
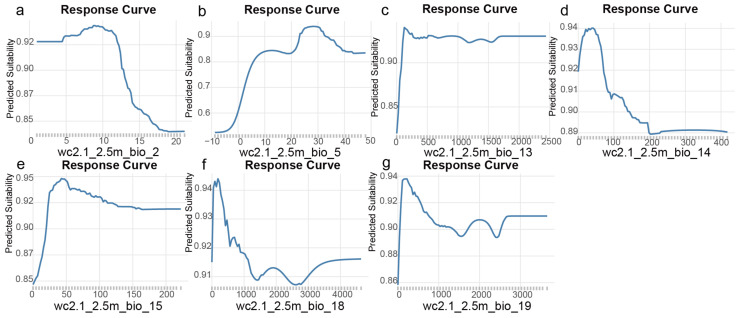
Response curves of the other seven environmental factors affecting the global suitable habitat of *L. camara.* (**a**) Mean diurnal temperature range (bio2), (**b**) max. temperature of warmest month (bio5), (**c**) precipitation of wettest month (bio13), (**d**) precipitation of driest month (bio14), (**e**) precipitation seasonality (bio15), (**f**) precipitation of warmest quarter (bio18), and (**g**) precipitation of coldest quarter (bio19).

## Data Availability

The original contributions presented in this study are included in the article. Further inquiries can be directed to the corresponding authors.

## Appendix A

**Table A1:** Single-model algorithm evaluation.

Algorithm	Evaluation Method	Cutoff	Sensitivity	Specificity	Calibration Value	Validation Value
ANN	AUC	404.75	91.45	80.96	0.90	0.90
ANN	TSS	404.33	91.49	80.90	0.72	0.72
CTA	AUC	497.50	93.40	89.17	0.95	0.94
CTA	TSS	497.00	93.40	89.17	0.83	0.81
FDA	AUC	507.00	93.17	88.02	0.96	0.96
FDA	TSS	517.00	93.04	88.13	0.81	0.81
GAM	AUC	488.67	94.34	89.78	0.97	0.97
GAM	TSS	485.00	94.39	89.70	0.84	0.84
GBM	AUC	521.50	93.93	89.01	0.97	0.97
GBM	TSS	523.33	93.87	89.04	0.83	0.82
GLM	AUC	517.83	91.90	87.42	0.95	0.95
GLM	TSS	516.67	91.93	87.35	0.79	0.80
MARS	AUC	483.67	93.67	88.79	0.97	0.97
MARS	TSS	498.33	93.33	89.09	0.82	0.82
MAXENT	AUC	29.00	92.71	88.42	0.97	0.96
MAXENT	TSS	33.50	91.95	89.03	0.81	0.81
RF	AUC	550.00	99.51	99.83	1.00	0.98
RF	TSS	550.50	99.51	99.83	0.99	0.88
SRE	AUC	500.00	63.23	91.02	0.77	0.77
SRE	TSS	495.00	63.23	91.02	0.54	0.54

**Table A2:** Integrated model algorithm evaluation.

Ensemble Method	Evaluation Method	Cutoff	Sensitivity	Specificity	Calibration Value
EMmean	AUC	560.50	95.38	92.90	0.987
EMmean	TSS	557.00	95.47	92.79	0.882
EMca	AUC	500.00	95.50	92.10	0.972
EMca	TSS	495.00	95.50	92.10	0.876
EMwmean	AUC	566.50	95.33	93.13	0.987
EMwmean	TSS	567.00	95.33	93.13	0.884
